# Strain-Independent Increases of Crystallin Proteins in the Retina of Type 1 Diabetic Rats

**DOI:** 10.1371/journal.pone.0082520

**Published:** 2013-12-13

**Authors:** Erich A. Heise, Lauren M. Marozas, Sean A. Grafton, Katelyn M. Green, Stefanie J. Kirwin, Patrice E. Fort

**Affiliations:** 1 Department of Ophthalmology and Visual Sciences, University of Michigan, Ann Arbor, Michigan, United States of America; 2 Biological Science, Allergan Incorporated, Irvine, California, United States of America; Faculty of Medicine University of Leipzig, Germany

## Abstract

Diabetic retinopathy is the leading cause of vision loss in working-age individuals in the United States and is expected to continue growing with the increased prevalence of diabetes. Streptozotocin-induced hyperglycemia in rats is the most commonly used model for diabetic retinopathy. Previous studies have shown that this model can lead to different inflammatory changes in the retina depending on the strain of rat. Our previous work has shown that crystallin proteins, including members of the alpha- and beta/gamma-crystallin subfamilies, are upregulated in the STZ rat retina. Crystallin proteins have been implicated in a number of cellular processes, such as neuroprotection, non-native protein folding and vascular remodeling. In this current study, we have demonstrated that unlike other strain-dependent changes, such as inflammatory cytokines and growth factor levels, in the STZ rat, the protein upregulation of crystallins is consistent across the Brown Norway, Long-Evans and Sprague-Dawley rat strains in the context of diabetes. Taken together, these data illustrate the potential critical role played by crystallins, and especially alpha-crystallins, in the retina in the context of diabetes.

## Introduction

Diabetes mellitus is a growing health concern as well as economic burden worldwide, especially due to the difficult management of its associated complications. Diabetic retinopathy (DR), which affects nearly all patients with more than 20 years of the disease, is the leading cause of blindness in working-age individuals in the United States. Streptozotocin (STZ)-induced diabetes in rats is the most common experimental model in the exploration of diabetic retinopathy. This model mimics human diabetes through the destruction of the β-cells in the pancreas, which leads to hypoinsulinemia and hyperglycemia [1]. Numerous studies have demonstrated commonalities between this animal model and the human pathology, including neuronal cell loss, glial cell activation, increased vascular permeability and inflammation [2]–[7]. It has also been shown, however, that different rat strains exhibit different responses to hyperglycemia and other insults to the retina [8]–[10]. The three most commonly used strains of rats used in diabetic retinopathy studies are Brown Norway (BN), Long-Evans (LE), and Sprague-Dawley (SD), the latter of which lacks pigmentation, excluding it from most studies examining visual function [11]. A study by Kirwin et al. reported that cytokine upregulation, a marker of inflammation, was observed in the retina of diabetic SD rats, but not LE or BN strains after diabetes induction by STZ. Eotaxin (CCL11), macrophage colony-stimulating factor (M- CSF), monocyte chemoattractant protein-1 (MCP-1; CCL-2), and MCP-3 (CCL-7) were all significantly upregulated only in the SD strain. This study also demonstrated some strain selectivity regarding the extent and variability of the effect of diabetes on growth factors levels such as vascular endothelial growth factor (VEGF) and fibroblast growth factor 2 (FGF-2; [12]). This study was the first to specifically demonstrate that some of the effects observed or associated with diabetes were strain dependent.

Crystallin proteins constitute a family of proteins containing similar structural domains. While they were primarily characterized in the lens and shown to play critical roles in maintaining its transparency, they have recently been shown to be of interest in other tissues due to their involvement in different cell and tissue function in normal and disease conditions. These proteins have been divided in 2 subfamilies, alpha- and beta/gamma-crystallin proteins. Alpha-crystallins are members of the small heat shock proteins (Hsp) family. We and others have previously shown that alpha-crystallins are upregulated in rodent models of type 1 and type 2 diabetes [13]–[15], and it has been suggested that this increased expression is an adaptive mechanism to protect retinal neurons from metabolic stresses. While alpha-crystallins have primarily been studied in the context of their chaperone function, they have also been implicated in the regulation of other cellular aspects such as inflammation, metabolism and cell survival [13], [16], [17]. We have also shown that members of the beta/gamma-crystallin subfamily were upregulated in rodent models of type 1 diabetes [13], [14]. Recent studies suggest that the upregulation of those proteins could reflect their involvement in different aspects of the pathogenesis of diabetic retinopathy including vascular remodeling (BetaA3/A1, [18]) and neuronal cell dysfunction and death (BetaB2, [19]). Our previous studies in the STZ rat model have been carried out in the non-pigmented Sprague-Dawley rat strain. The current study was performed to test the conservation of the crystallin induction in retina during diabetes as a function of genetic background. In this study, we analyzed the expression and regulation of crystallins, as well as the phosphorylation pattern of alpha-crystallins in BN, LE and SD diabetic and age-matched control rats.

## Materials and Methods

### Ethics Statement

All experiments were conducted in accordance with the Association for Research in Vision and Ophthalmology Resolution on the Care and Use of Laboratory Animals and these studies were specifically approved by the University of Michigan (UCUCA #10463) animal care and use committees.

### Induction of Diabetes and Tissue Collection

Age-matched male Brown Norway, Long-Evans and Sprague-Dawley rats (Charles River, MA) were housed under a 12 h light/dark cycle with free access to a standard rat chow and water. All experiments were conducted in accordance with the Association for Research in Vision and Ophthalmology Resolution on the Care and Use of Laboratory Animals. Diabetes was induced by intraperitoneal injection of streptozotocin (STZ) (65 mg/kg; Sigma, St. Louis, MO) dissolved in sodium citrate buffer, pH 4.5, and control rats received equivalent volumes of buffer alone as described previously [2]. STZ-injected rats were considered diabetic when exhibiting blood glucose levels >13.9 mmol/l (250 mg/dl) within 5 days after diabetes induction (One-Touch meter; Lifescan, Milpitas, CA or Ascencia control system; Bayer, Tarrytown, NY). The 4 and 12 weeks diabetes duration studies were chosen because they lead to increased neuronal cell death, microvascular leakage, astrocyte defects, microglial cell activation, and impaired insulin receptor signaling [2]–[5], [20]. Retinas were immediately frozen in liquid nitrogen and stored at −80°C until analysis. For RNA extraction retinas were placed in 200 µl RNA stabilizer (RNAlater, Ambion, Austin, TX) and incubated at 4°C for 24 h before removal of excess liquid and transfer to −80°C.

### RNA Isolation and Real Time PCR

Total RNA was isolated by homogenizing frozen samples in 1 ml lysis reagent (Qiazol, Qiagen, Valencia, CA) using Tissue – Tearor (Biospec, Bartlesville, OK) followed by extraction (RNeasy Mini Kit, Qiagen). After RNA quality assessment (RNA 6000 Nano Kir, Agilent Technologies, Waldbronn, Germany) and quantification by spectrophotometry, reverse transcription was performed on 1 µg of RNA using 25 mM MgCl_2_, 10 mM dNTP mixture, 25 U RNasin, and 15 U AMV reverse transcriptase in reverse transcription buffer (all Promega, Madison, WI) in 20 µL for 20 minutes at 42°C, followed by a 5-minute denaturing step at 95°C and 5 minutes at 4°C. After reverse transcription, ddH_2_O was added to a final volume of 200 µL. Quantitative PCR was performed on the 7900HT Sequence Detection System (Applied Biosystems, Foster City, CA), using the following primer sets (*Taq*man, ABI) for Actb (ID:Rn00667869_m1), Cryaa (ID:Rn00561064_m1), Cryab (ID:Rn00564026_m1), Cryba1 (ID:Rn01496018_m1), Cryba2 (ID:Rn00517617_m1), Cryba4 (ID:Rn00581656_m1), Crybb1 (ID:Rn00564028_m1), Crybb2 (ID:Rn00564035), Crybb3 (ID:Rn00581661), Crygb (ID:Rn02110197_s1), Crygc (ID:Rn02110370_s1), Crygd (ID:Rn01441464), Crym (ID:Rn00588604). Gene expression was normalized and converted to a linearized value by the following formula: unit  = 1.8 ^∧^ (Ct_actb_ − Ct_gene*x*_) ×100.

### Immunoblot Analysis

Retinas were homogenized by sonication in immunoprecipitation (IP) buffer as previously described [21]. Protein concentrations were measured with the BCA (bicinchoninic acid) protein assay (Thermo Fisher, Rockford, IL), and all samples were adjusted for equal protein concentration. Retinal lysates were used for immunoblot analysis using the following antibodies: pan-specific antibodies directed against α-, β-, and γ-crystallins (generously provided by Dr. Samuel Zigler), specific antibodies against αA-crystallin (Santa Cruz Biotech, Santa Cruz, CA), αB-crystallin (Abcam, Cambridge, MA), against alphaB-crystallin Phospho S19, Phospho S45 and Phospho S59 (Enzo Life Science, Farmingdale, NY). Immunoblots were performed as described previously but using 4–12% NuPAGE gels [22] and MES buffer following the manufacturer's instructions. Results were normalized by reprobing the same membrane using an antibody against actin (EMD Millipore, Billerica, MA).

### Statistical Analysis

For all immunoblot experiments, the data were normalized to the actin signal as control before analysis. Fisher's T-test models, adjusted for the replication of the experiment, were fit to the data to assess differences between diabetic and control rats of each strain. The mean ±SEM and statistically significant differences are reported. Statistical analysis (2 ways analysis of variance) of the variability between strains was performed and failed to show any statistical differences in either control or diabetic conditions.

## Results

**Figure 1:**
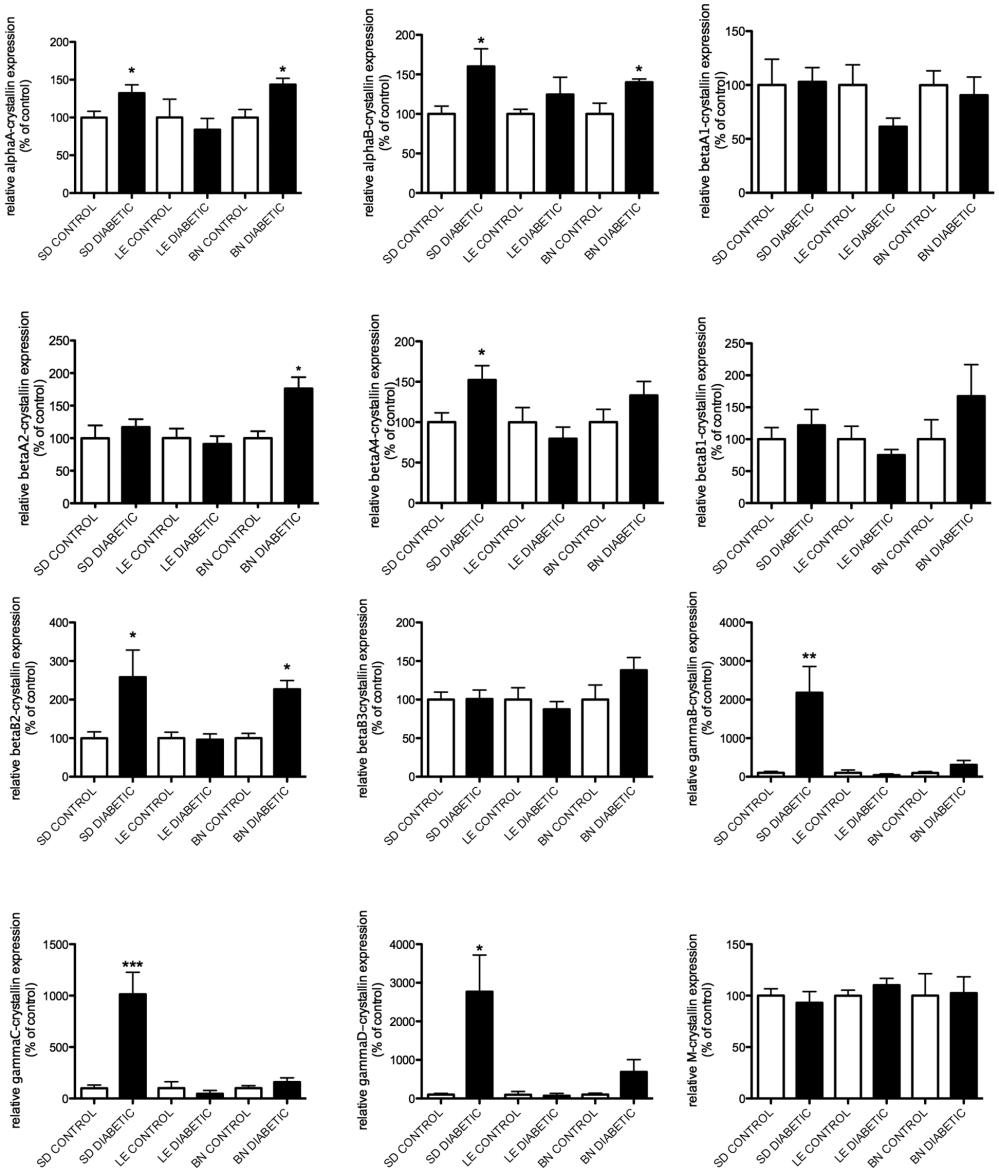
Gene expression analysis of crystallins after 4 weeks of diabetes. mRNA levels of crystallin genes (alpha-, beta- and gamma-) were assessed by Real Time PCR using gene-specific primers and probes 4 weeks after the onset of diabetes in Sprague Dawley (SD), Long Evans (LE) and Brown Norway (BN) rats. A representative graphic representation of the corresponding quantification is presented for alphaA-, alphaB-, betaA1-, betaA2-, betaA4-, betaB1-, betaB2-, betaB3-, gammaB-, gammaC-crystallin, gammaD- and M-crystallin mRNA respectively (A-L). *Significantly different from control [*P*<0.05].

**Figure 2:**
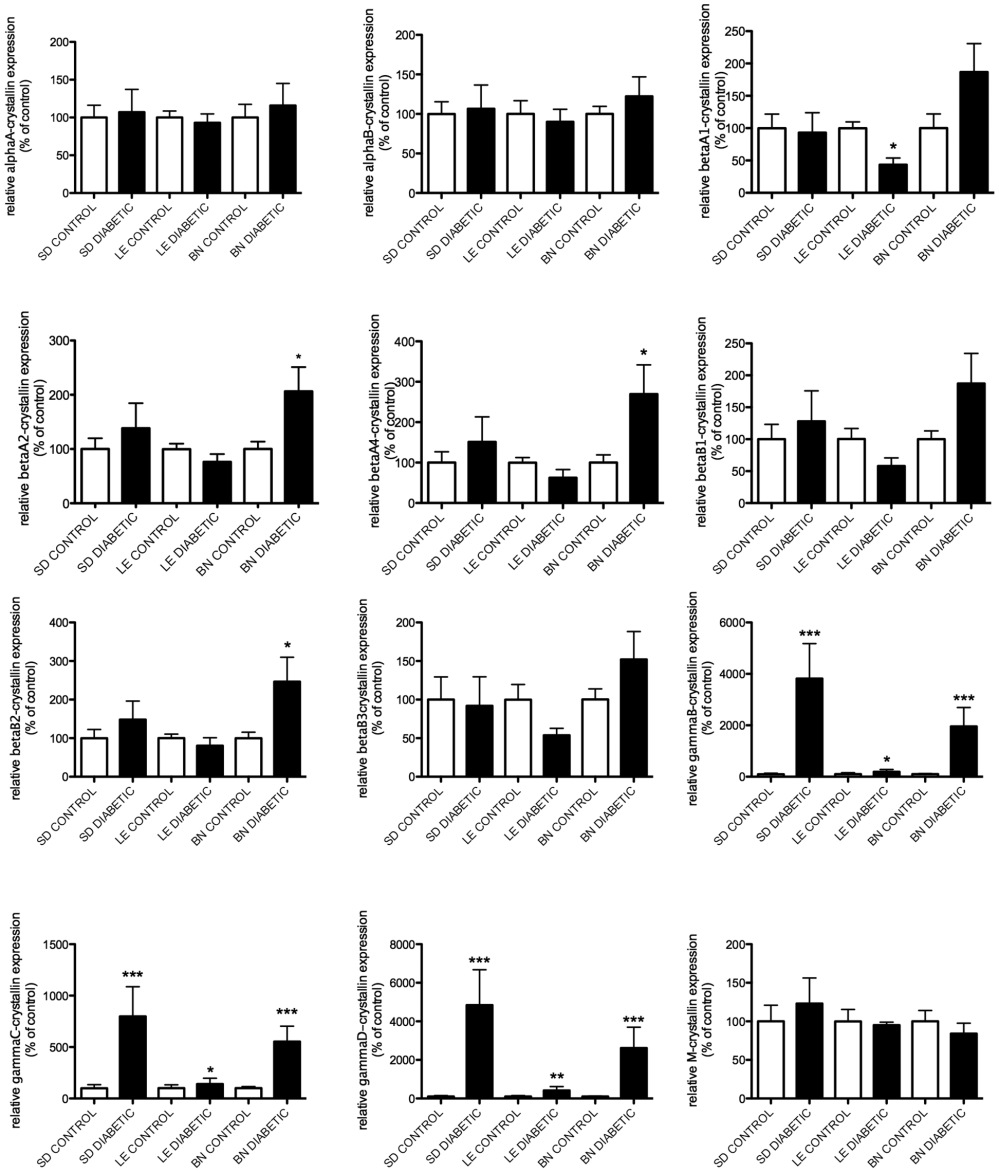
Gene expression analysis of crystallins after 3 months of diabetes. mRNA levels of crystallin genes (alpha-, beta- and gamma-) were assessed by Real Time PCR using gene-specific primers and probes 12 weeks after the onset of diabetes in Sprague Dawley (SD), Long Evans (LE) and Brown Norway (BN) rats. A representative graphic representation of the corresponding quantification is presented for alphaA-, alphaB-, betaA1-, betaA2-, betaA4-, betaB1-, betaB2-, betaB3-, gammaB-, gammaC-crystallin, gammaD- and M-crystallin mRNA respectively (A-L). *Significantly different from control [*P*<0.05].

Gene expression levels of members of the alpha- and beta/gamma-crystallin families were assessed by Real Time PCR at 4 and 12 weeks after the onset of diabetes (Fig. 1 & 2). At 4 weeks, a slight increase in alphaA- and alphaB-crystallin mRNA levels was observed in the diabetic rats of the SD and BN strains compared to the mRNA levels of their control littermates. A much larger effect was detected for members of the gamma-crystallin family. GammaB-, gammaC- and gammaD-crystallins displayed significant upregulation in diabetic rats of the SD strain and while a similar trend was seen in the BN diabetic rats at 4 weeks (Figure 1), this did not reach statistical significance. At 12 weeks, gamma-crystallin mRNA levels were further enhanced in diabetic SD rats while both BN and LE diabetic rats displayed a significant upregulation in gammaB-, gammaC- and gammaD-crystallin mRNA levels. Interestingly, the mRNA levels of alphaA- and alphaB-crystallin in the diabetic SD rats no longer displayed a significant upregulation compared to their control littermates (Figure 2). These data are consistent with our previous results in SD rats showing that diabetes leads to an increased level of alpha-crystallin proteins without a correlated increase in their mRNA expression [14]. Conversely gamma-crystallin induction by diabetes is transcriptionally regulated in SD rats. While profiles of transcription of all crystallin proteins were similar in all three strains tested, beta-crystallin transcripts were only affected by 12 weeks of diabetes in BN rats (Figure 2). These data suggest that there might be more transcriptional regulation of crystallin protein expression in BN than other rat strains tested in this study.

**Figure 3:**
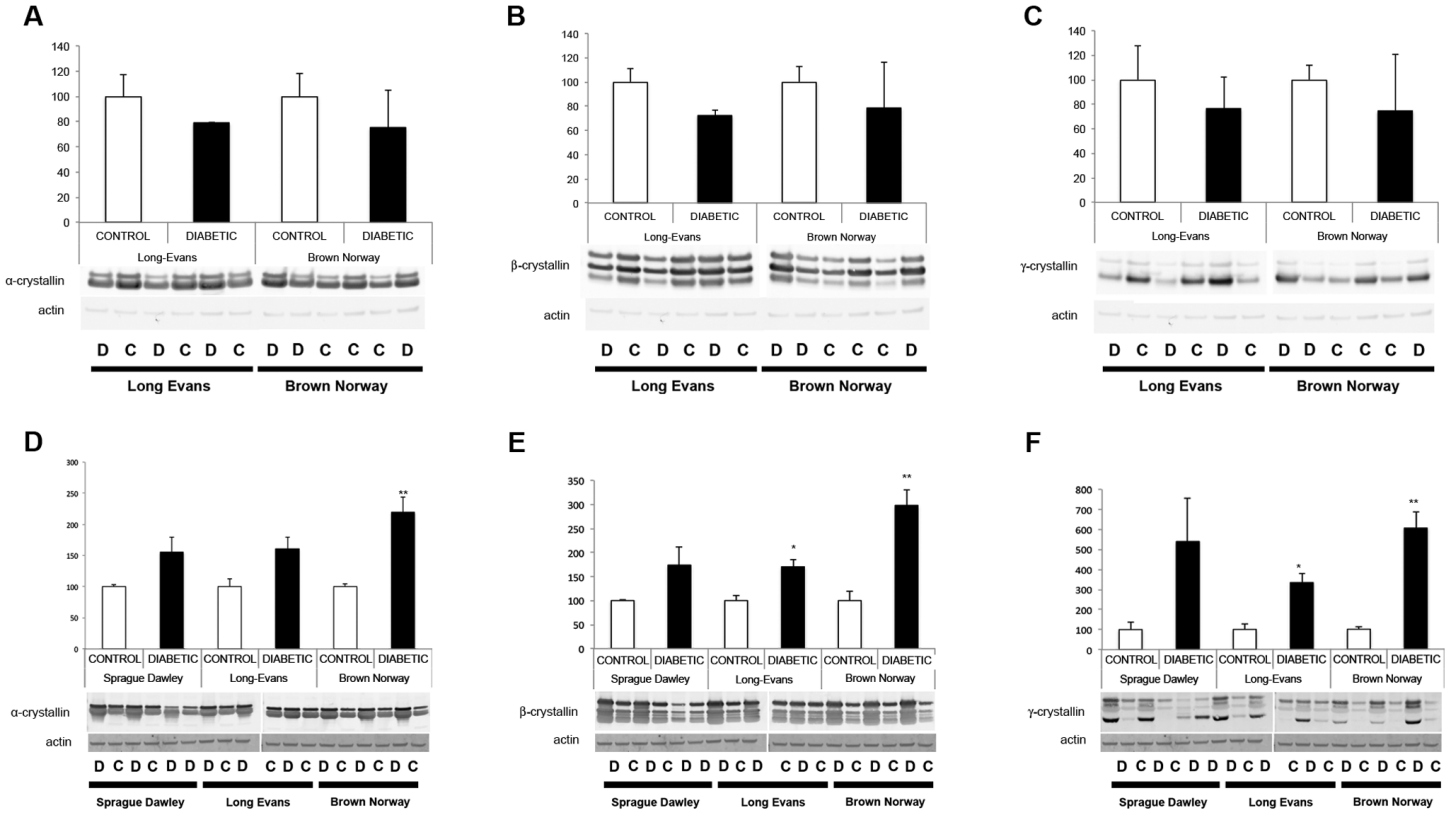
Protein expression analysis of alpha-, beta- and gamma-crystallins during diabetes. Expression levels of crystallin proteins (alpha-, beta- and gamma-) were assessed by Western blot analysis using pan-specific antibodies raised against alpha- (A, D), beta- (B, E) and gamma- (C, F) crystallin proteins respectively, 4 weeks after the onset of diabetes (A–C) in Long Evans (LE) and Brown Norway (BN) rats. Expression levels were also assessed at 12 weeks (D–F) in Sprague Dawley (SD), Long Evans (LE) and Brown Norway (BN) rats. Crystallin expression is normalized to actin levels and relative to the expression of the control littermates. Representative immunoblots and graphic representation of the corresponding quantification of the main products are presented for alpha-, beta- and gamma-crystallin proteins respectively. C, control; D, diabetic. *Significantly different from control [*P*<0.05].

**Figure 4:**
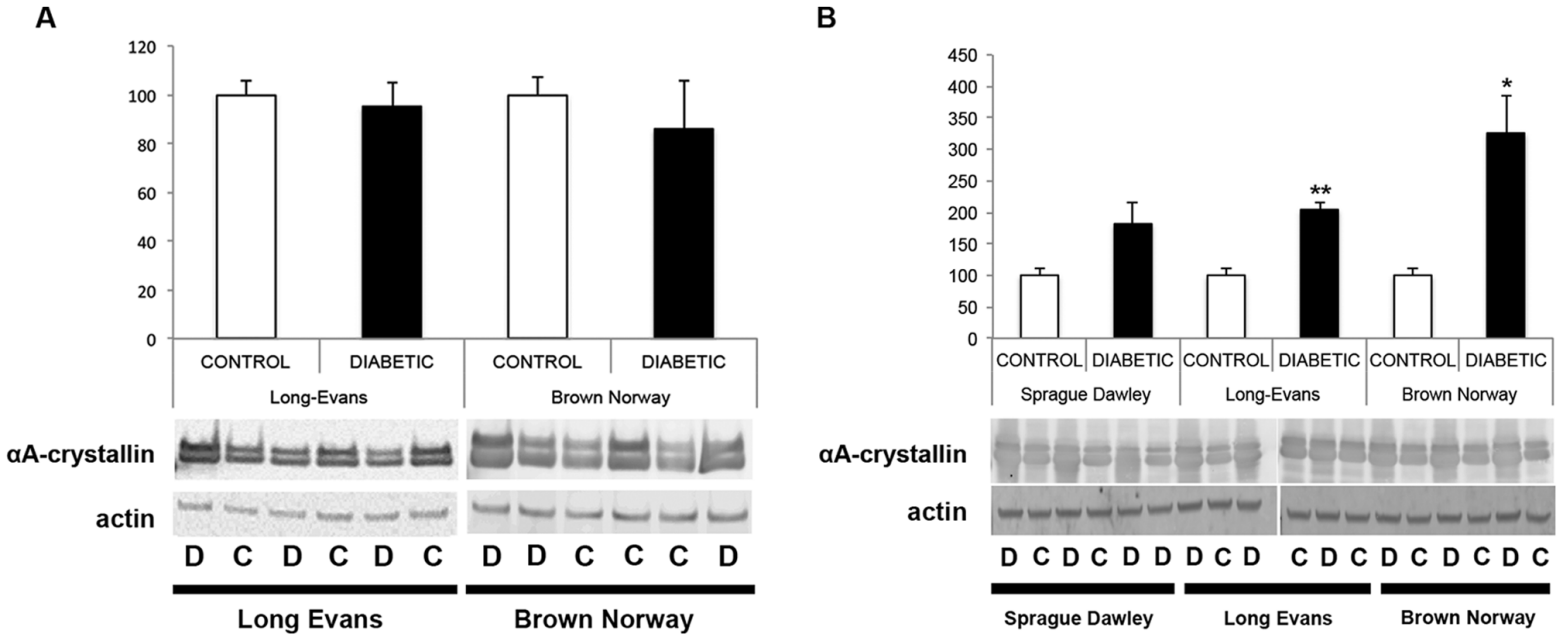
Protein expression analysis of alphaA-crystallin during diabetes. Expression levels were assessed with Western blot analysis using specific antibodies raised against alphaA-crystallin proteins 4 weeks after the onset of diabetes (A) in Long Evans (LE) and Brown Norway (BN) rats. Expression levels were also assessed at 12 weeks (B) in Sprague Dawley (SD), in Long Evans (LE) and Brown Norway (BN) rats. Representative immunoblots and graphic representation of the corresponding quantification of the major bands are presented. C, control; D, diabetic. *Significantly different from control [*P*<0.05].

**Figure 5:**
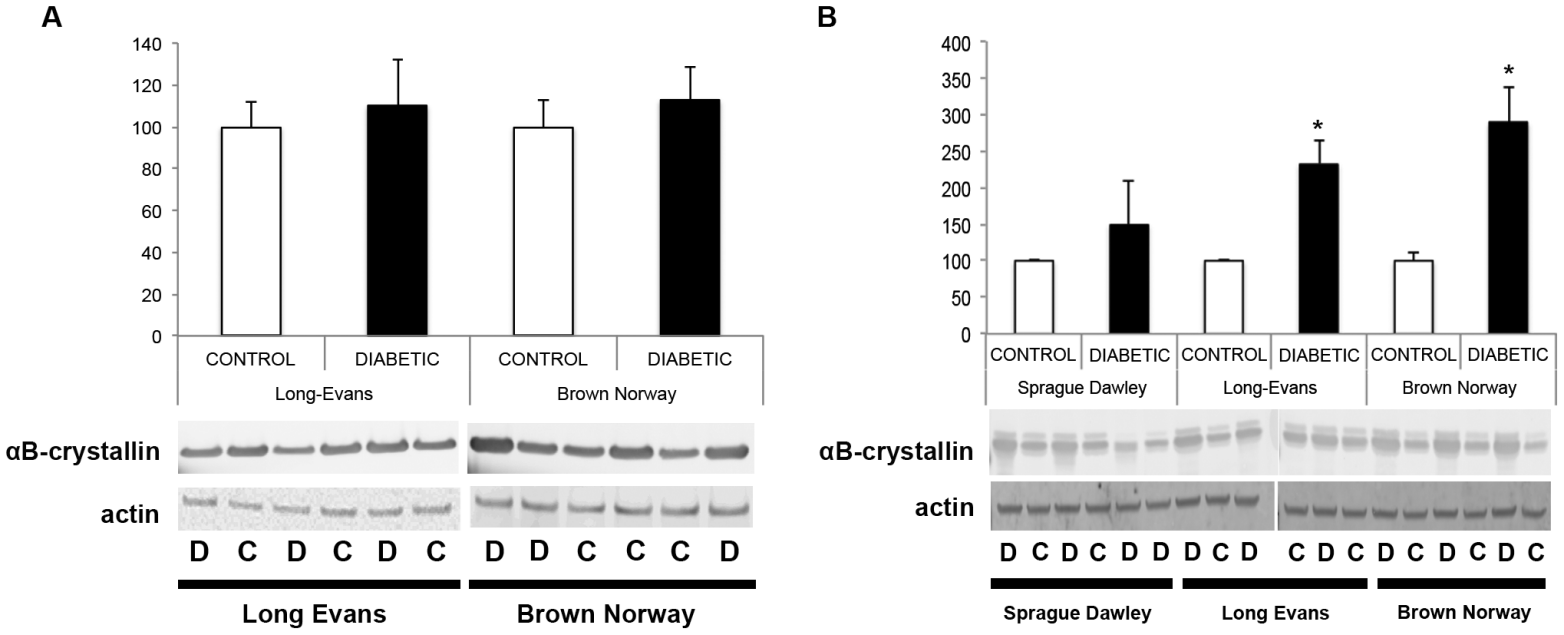
Protein expression analysis of alphaB-crystallin during diabetes. Expression levels were assessed with Western blot analysis using specific antibodies raised against alphaB-crystallin proteins 4 weeks after the onset of diabetes (A) in Long Evans (LE) and Brown Norway (BN) rats. Expression levels were also assessed at 12 weeks (B) in Sprague Dawley (SD), in Long Evans (LE) and Brown Norway (BN) rats. Representative immunoblots and graphic representation of the corresponding quantification of the major bands are presented. C, control; D, diabetic. *Significantly different from control [*P*<0.05].

**Figure 6:**
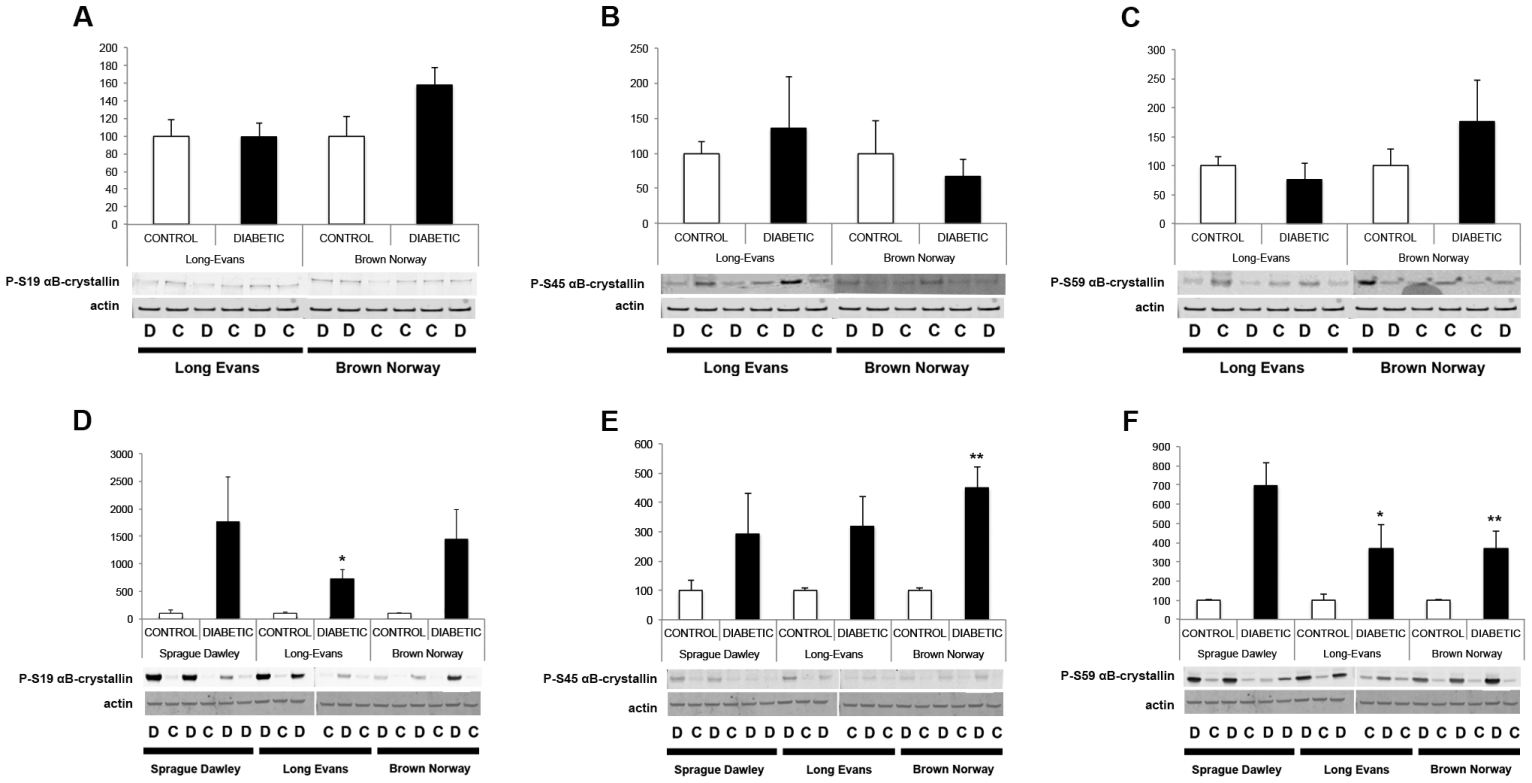
Phosphorylation analysis of alphaB-crystallin during diabetes. The expression of alphaB-crystallin protein phosphorylated at serines 19 (A, D), 45 (B, E) and 59 (C, F) were assessed by Western blot analysis using specific antibodies raised against alphaB-crystallin protein phosphorylated at those sites 4 weeks after the onset of diabetes (A–C) in Long Evans (LE) and Brown Norway (BN) rats. Expression levels were also assessed at 12 weeks (D–F) in Sprague Dawley (SD), in Long Evans (LE) and Brown Norway (BN) rats. Representative immunoblots and graphic representation of the corresponding quantification of the major bands are presented. C, control; D, diabetic. *Significantly different from control [*P*<0.05].

We previously showed that crystallin protein expression is primarily regulated post-transcriptionally in SD rats; thus, crystallin expression patterns were assessed by immunoblot analysis 4 and 12 weeks after the onset of diabetes (Figure 3). At 4 weeks after the onset of diabetes, pan-specific antibodies confirmed that as in the SD rats, alpha-, beta- and gamma-crystallin protein levels were unaltered in the LE and BN diabetic rats compared to their control littermates. At 12 weeks, all three subgroups of crystallin proteins were significantly upregulated in LE and BN diabetic rats. Since we already extensively studied and published the effect of diabetes on crystallins in the SD rat strain, few samples were used and no statistical analysis was performed for this rat strain, but similar trends were observed and support our previously published results [14]. Furthermore, detailed analysis of alpha-crystallin protein expression was performed using specific antibodies raised against alphaA- and alphaB-crystallin proteins. At 4 weeks of diabetes, no differences were detected between diabetic rats and their littermate controls in either the LE or BN strains. At 12 weeks, there was a significant upregulation of both alphaA- and alphaB-crystallin proteins in LE and BN diabetic rats (Figures 4 & 5). Despite this consistent upregulation of alpha-crystallin proteins, increased cell death has been reported, suggesting that their functions could be inhibited. Post-translational modifications, such as glycation, phosphorylation and cleavage, have been implicated in the downregulation of the chaperone activity, or non-native protein refolding, of crystallins due to changes in the surface charges [15], [23]. Therefore, we also assessed the phosphorylation profile of alphaB-crystallin protein on the specific serine residues, S19, S45 and S59, by immunoblot analysis. At the 4 week time-point, LE and BN strains displayed no consistent pattern of phosphorylation of any of the three serine residues on alphaB-crystallin proteins. However, at 12 weeks, diabetic rats from all three strains, SD, LE and BN, displayed increased phosphorylation on all three serine residues S19, S45 and S59 of alphaB-crystallin when compared to their control littermates (Figure 6). These data strongly suggest that the chronic state of diabetes alters the functional state of alpha-crystallins.

## Discussion

We previously showed that several members of the crystallin protein family were highly upregulated in streptozotocin-induced Sprague-Dawley diabetic rats [14], [15], [24]. We are now reporting that this upregulation, at least at the protein level, is a hallmark of diabetes in the retina as it is conserved across species and across different rat strains. Indeed, we are reporting here that this strong upregulation of crystallin proteins is conserved across BN, LE and SD, three of the most commonly used strains of rats in the diabetes field, while other changes in the context of diabetes, including aspects of the inflammatory response and growth factor signaling, and analyzed in either the same retinas (RNA) or the contralateral retina from the same animals (proteins), are highly strain dependent [12]. A well-known feature of the alpha-crystallin subfamily is their chaperone function, which is the ability to refold nonnative proteins to prevent their irreversible aggregation. This chaperone function plays a vital role in the context of stress and may explain the conserved upregulation of alpha-crystallins in the retina during diabetes. We performed this study to test our hypothesis that despite strain selectivity regarding growth factor signaling and inflammatory responses in the context of diabetes, crystallin induction and upregulation is conserved across different rat strains supporting its role in the adaptive mechanisms taking place in the retina during diabetes.

Our previous data suggested that alpha- and beta-crystallins are mainly regulated at the translation level while gamma-crystallins are regulated at the transcription level [14]. These observations are consistent with the dramatic upregulation of the gamma-crystallin mRNA in diabetic rats at 4 and 12 weeks reported in the current study. Alpha- and beta-crystallin mRNA showed less changes at the messenger level. Additionally, as assessed by Western blot at 12 weeks, alpha- and beta-crystallins showed upregulation in all three strains of diabetic rats, confirming regulation at the post-transcriptional level. Interestingly, diabetic BN rats showed a greater disparity in mRNA expression levels when compared to their controls of several crystallin proteins, suggesting that BN rats may be regulated to a higher degree at the transcription level than the other two strains, SD and LE.

In this study, we demonstrated that alphaA- and alphaB-crystallin were upregulated across all three strains suggesting they play a key role in the impact of diabetes on the retina. Such an increase of alpha-crystallin proteins could reflect an adaptive response of the tissue to counteract pro-apoptotic mechanisms. We previously showed that alphaA- and alphaB-crystallin could protect retinal neurons from acute stress-induced cell death [13]. Despite the upregulation of alphaA- and alphaB-crystallin, cell death still occurs in the context of diabetes [3] suggesting that despite their protective role in the retina during transient stress episodes, increased levels of alpha-crystallins cannot completely prevent retinal cell death associated with the chronic stress of diabetic retinopathy. Crystallin proteins are highly post-translationally modified in the retina [14], [25]. In this study, we have demonstrated that alphaB-crystallins were highly phosphorylated on serines 19, 45 and 59 in the context of diabetes across SD, BN and LE rat strains. Phosphorylation of alpha-crystallins leads to a change in surface charges, which, in turn, affects their interactions with other proteins. Subsequently, these affected protein-protein interactions alter the chaperone function of alpha-crystallins [23]. While we previously showed that levels of activated Bax are increased in the retina of SD diabetic rats [13], alphaB-crystallin has been shown to sequester proapoptotic members of the Bcl-2 family, Bax and Bcl-Xs, in the cytoplasm, maintaining mitochondrial integrity and preventing apoptosis [26]. Increased phosphorylation of alphaB-crystallin on serine 59 has been suggested to reduce its anti-apoptotic function through sequestration of Bcl-2 in the cytosol [27]. Similarly, interaction of alpha-crystallin and Bax, which is reduced in diabetes, may also be affected by increased phosphorylation, which could, in turn, explain the increased apoptosis observed in the diabetic retina. Interestingly, during experimental uveitis, overexpression of alphaA-crystallin has a neuroprotective effect on photoreceptor cells through its interaction with cytochrome c and procaspase-3, preventing the activation of the latter [28]. This finding suggests that even in a chronically stressed environment, alpha-crystallin can still play a protective role, which might only be transient unless controlled by protecting it from inhibitory post-translational modifications or by using only smaller fragments of the proteins [29].

In the present study, we also report an upregulation of members of the beta- and gamma-crystallin family in diabetic rats in all three strains. Double-labeling experiments with isolectin B4 and beta- and gamma-crystallin antibodies have shown that beta- and gamma-crystallin expression were concentrated in the astrocytes surrounding the vasculature of the retained hyaloid artery in Nuc1 rats, suggesting their involvement, together with VEGF, in vascular remodeling [30]. Diabetic retinopathy remains primarily diagnosed as a microvascular complication of diabetes; alteration of the protein level of beta- and gamma-crystallin could reflect their implication in the microvascular changes, such as retinal venular widening, which leads to loss of visual acuity and ultimately blindness [31]. Further studies are needed to test this hypothesis but recent studies have shown that betaA3/A1-crystallin is expressed differentially in various ocular tissues and may play a role in vascular remodeling [32]. Additionally, we have shown gamma-crystallin upregulation in ganglion cells, more specifically in their axons, in the context of diabetes [14]. BetaB2-crystallin has been shown to possess neurite-promoting activity in cultures of retinal stripes [19]. Likewise, the overexpression of betaB2-crystallin by neuronal progenitor cells (NPC) has been shown to exert beneficial effects on the vitreoretinal compartment [33]. Gastinger *et al.* have demonstrated a loss of retinal ganglion cells as well as abnormal axonal and dendritic swelling in the surviving retinal ganglion cells within three months after the onset of diabetes in rats [34]. Therefore, betaB2- and gamma-crystallins could play a pivotal role in the alterations of ganglion cell anatomy, function and survival observed in diabetes conditions.

Altogether these data demonstrate a strain-independent conservation of the upregulation of crystallin proteins in the context of diabetes. This cross-strain conservation confirms the critical nature of the role played by crystallin proteins in the retinal tissue specific reaction to diabetes and the possible implication that proteins from this family could be playing in different aspects of the way this tissue handles diabetes related stress and the development of diabetic retinopathy. Evidences in the literature points towards some crystallins, such as betaB2 and alphaA, serving a role in the protection of retinal ganglion cells, while other crystallins, such as betaA3/A1 and alphaB, may serve a role in the regulation of vascular remodeling of the retina. Further studies are needed to fully elucidate the role of crystallins in the pathophysiology of diabetic retinopathy.
